# Clinical Versatility of the Facility-Equator Implant System as Mandibular Overdenture Retainers

**DOI:** 10.1155/2020/8823547

**Published:** 2020-11-29

**Authors:** Anna Paula da Rosa Possebon, Alessandra Julie Schuster, Amália Machado Bielemann, Ana Paula Pinto Martins, Samille Biasi Miranda, Otacílio Luiz Chagas-Júnior, Luciana de Rezende Pinto, Fernanda Faot

**Affiliations:** ^1^Graduate Program in Dentistry, School of Dentistry, Federal University of Pelotas, RS, Brazil; ^2^Department of Restorative Dentistry, School of Dentistry, Federal University of Pelotas, RS, Brazil; ^3^School of Dentistry, Federal University of Pelotas, RS, Brazil; ^4^Department of Oral and Maxillofacial Surgery and Maxillofacial Prosthodontics, School of Dentistry, Federal University of Pelotas, RS, Brazil

## Abstract

The use of mandibular overdentures (MO) for the rehabilitation of totally edentulous individuals with limited bone availability is widespread and has proven clinical success. Narrow diameter implants (NDI) are available on the market as MO retainers to solve problems related to limited bone availability and bone thickness, providing a low-cost, minimally invasive treatment option. This technique evolved over the years, and changes frequently involved the number of implants used as MO retainers, as the adoption of a smaller number of implants can generate biomechanical disadvantages, contributing to the increased stress in peri-implant tissues, which may accelerate marginal bone loss (MBL), in addition to reducing masticatory capacity and satisfaction with rehabilitation. Some studies pointed out that the use of 3 or more implants as MO retainers improves the biomechanics. Thus, the objective of this study was to report 3 different clinical cases where 3 or more NDI were adopted to retain mandibular overdentures in association with diverse loading protocols: (i) 3 implants adopting conventional loading, (ii) 4 implants using progressive loading, and (iii) 4 implants with hybrid loading. The case with 4 implants and progressive loading showed a slight worsening of masticatory function at 1 year, in addition to a more pronounced MBL compared to other cases, but with improvements in satisfaction and oral health-related quality of life. Thus, NDI can be used as MO retainers with predictability and clinical success, using different numbers of implants and loading protocols.

## 1. Introduction

Complete denture wearers often report complaints related to lack of stability and retention, especially of the mandibular prostheses [1]. These complaints are mainly related to bone availability and residual ridge morphology, which are dependent on the severity of bone resorption and the time of edentulism [1]. These factors result in difficulties using mainly the mandibular prosthesis that directly affect quality of life and contribute to maintaining an impaired or deficient chewing pattern [2, 3].

According to the McGill consensus, mandibular overdentures (MO) retained by 2 implants should be the minimum treatment offered to this profile of patients, due to the benefits that this type of rehabilitation promotes, such as increased bite force, decreased discomfort during function, improvements in masticatory function, and neuromuscular control, thus ensuring greater quality of life and patient satisfaction [4]. Several MO retention systems are available and differ mainly in terms of whether or not they use implant splinting. Currently, nonsplinted systems are used more frequently, both in cases of conventional and immediate loading, ensuring treatment with high predictability and safety, in addition to easy handling by the patient and simplified maintenance routine [5, 6].

Narrow diameter implants (NDI) have become an alternative rehabilitation for patients with limited bone availability, especially for elderly patients, guaranteeing the rehabilitation of edentulous patients with residual ridge atrophy, who often could not undergo an extensive surgical procedure with prolonged healing time [7]. The Facility-Equator NDI implant system is available on the market since 2013 and consists of a small diameter implant (2.9 mm) with a 5° Morse cone connection based on frictional retention. Its prosthetic system uses a screwless button-type fitting system that is installed with the help of a hammer (Facility Equator attachment; transmucosal heights range from 1.5 to 4.5 mm) [8]. Previous studies that investigated the predictability and success of the Facility-Equator system [3, 8–11] show that the use of 2 implants leads to excellent clinical and functional performance for the rehabilitation of totally edentulous individuals. However, younger edentulous patients using MO retained by only 2 implants still complain about retention in the posterior region. In this sense, some studies show that the use of 3 or more implants as MO retainers would improve the biomechanics, since MO retained by 2 implants can still present the tendency to move in the anteroposterior direction. Thus, the use of a 3^rd^ or 4^th^ implant would guarantee greater retention and stability, in addition to reducing anteroposterior movement [12–14].

Thus, the objective of this study was to explore the effect of using different numbers of NDI (Facility-Equator system) to retain MO combined with various loading protocols in a series of clinical cases describing the predictability of this system by monitoring clinical, functional, and patient-centered outcomes after 1 year of use. The following combinations of implant numbers and type of occlusal loading were adopted in these 3 clinical cases: (i) 3 NDI with conventional loading, (ii) 4 NDI with progressive loading, and (iii) 4 NDI with hybrid loading.

## 2. Clinical Case Reports

First, all individuals that agreed to participate in this study were asked to sign an informed consent form. This case series reportfollowed the CARE Guidelines [15]. In all clinical cases, the individuals were nonsmokers, did not have systemic diseases, and were totally edentulous in the upper arch. In the mandible, 1 participant used complete dentures and 2 used removable partial dentures. All patients showed clinically limited bone thickness with Class IV mandibular ridge resorption according to Cawood and Howell's descriptive classification and experienced persistent lack of retention and stability of their mandibular prostheses [16], in addition to discomfort, pain, and poor masticatory efficiency that led them to stop using their removable mandibular prostheses.

**Figure 1:**
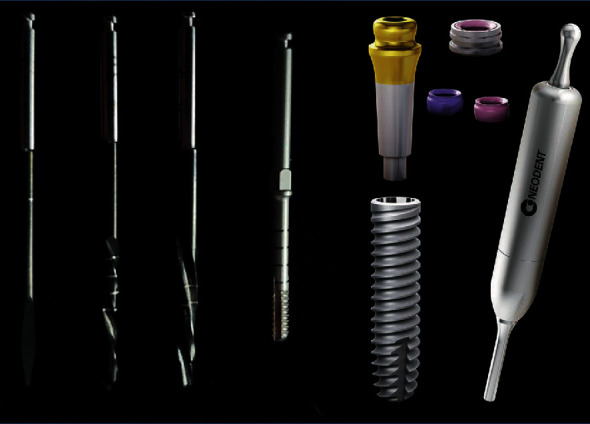
The Facility-Equator system. Sequence of drills used for installing the narrow diameter implant (Facility Initial Drill, Facility Twist Drill 2.0, Facility Drill 10, Facility); Facility implant (Ø 2.9 mm × 10,12,14 mm); Facility-Equator attachment, Equator O-ring with Cylinder, Equator O-ring (pink: less retention; purple: more retention), and Facility Abutment Placement Aid.

MO retained by NDI (Facility-Equator) was indicated for all patients; 3 or 4 implants were installed between the mental foramen, depending on the patients' preference, desired degree of retention, financial situation, and bone availability. The implant insertion torque determined the type of loading used in the 3 cases: (i) immediate loading was used when the implant insertion torque was ≥32 N, (ii) conventional loading when the insertion torque was <32 N, and (iii) hybrid loading was applied in cases where 4 implants were used and where at least 2 implants achieved the minimum insertion torque required for immediate loading, and the remaining cases were loaded 3 months after surgery. In all cases, implant installation followed the bone drilling protocol recommended by the manufacturer (Figure 1), adopting a minimally invasive technique with a soft tissue flap restricted to the implant area. The procedure was performed by an experienced surgeon (OLCJ). Equator-type attachment was installed with a hammer (Facility Abutment Placement Aid) designed for insertion of prosthetic components through impact (Figure 1). Postsurgical instructions included the use of medication ((i) antibiotic: amoxicillin 875 mg, 1 tablet every 12 hours for 7 days; for those allergic to penicillin, azithromycin 500 mg, 1 tablet every 24 hours for 5 days; (ii) anti-inflammatory: nimesulide 100 mg, 1 tablet every 12 hours for 4 days; (iii) analgesic: dipyrone 500 mg, 2 tablets every 6 hours in case of pain or fever; for those allergic to dipyrone, paracetamol 750 mg, 1 tablet every 6 hours in case of pain or fever). The patients were recommended to eat cold and pasty food in the first 48 hours and to rest and avoid intense physical exercise, spitting, sucking, and exposure to hot sun during the first 48 hours. The sutures were removed 10 days after surgery.  Table 1 lists the clinical information and characteristics of all patients in this case series.

### 2.1. Case 1

**Figure 2:**
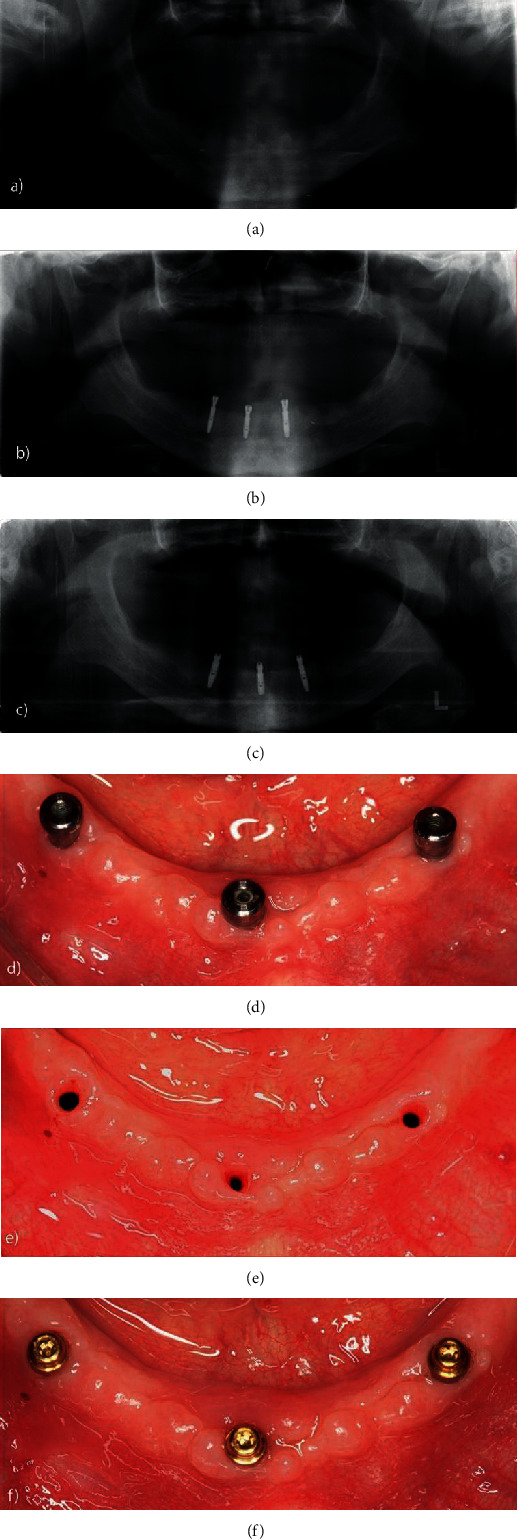
Panoramic X-rays: (a) preoperative, (b) immediately postoperative, and (c) 1 year postoperative; (d) peri-implant healing after 3 months; (e) tissue condition after removal of the healing caps; (f) installation of prosthetic attachment and adaptation of peri-implant tissues around the Equator attachment.

**Figure 3:**
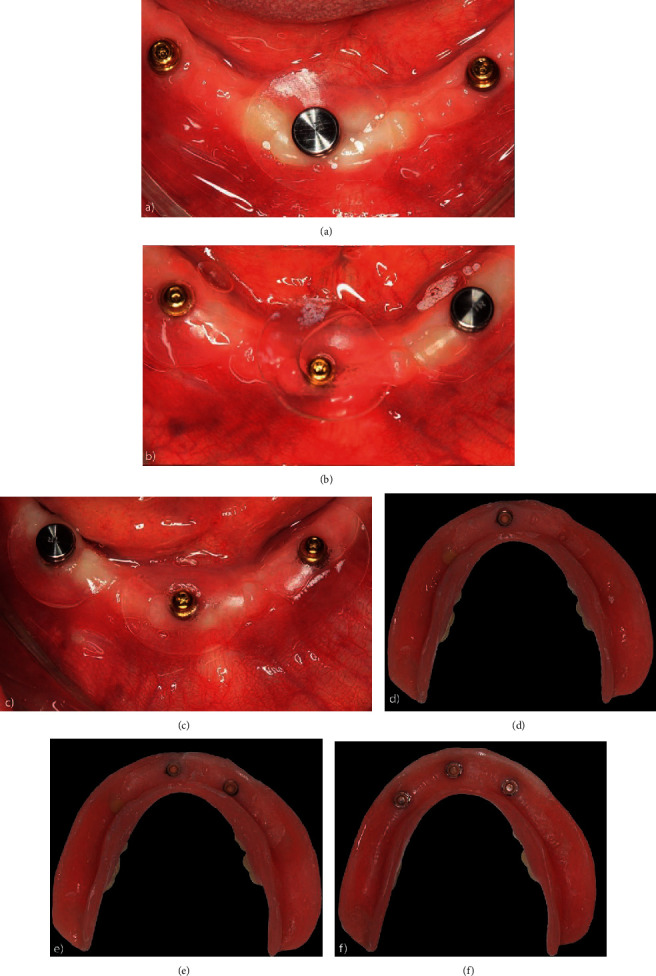
(a–c) Capture sequence for the O-rings with cylinders; (d) central Equator attachment capture; (e) left Equator attachment; (f) right Equator attachment capture.

The patient presented an ovoid mandibular ridge with a rounded asymmetric residual ridge and a medium muscle insertion. In this case, 3 implants were installed because the patient is younger and requested a higher degree of retention. The patient was unsatisfied with his prosthetic experience after extraction of the lower teeth, as he was unable to adapt to the immediate mandibular total prosthesis in the short period of edentulism (3 months). Conventional loading was applied since no implant achieved the minimum insertion torque of 32 N during installation.  Figure 2 shows the radiographic exams performed prior to surgery and those made in the immediate postoperative period and after 1 year (Figures 2(a)–2(c)). Figures 2(d)–2(f) illustrate the adaptation of peri-implant tissues around the healing caps immediately after replacement by Equatorattachments after 3 months of osseointegration.  Figure 3 shows the capture sequence of each O-ring with cylinder for the loading phaseseparately.

### 2.2. Case 2

**Figure 4:**
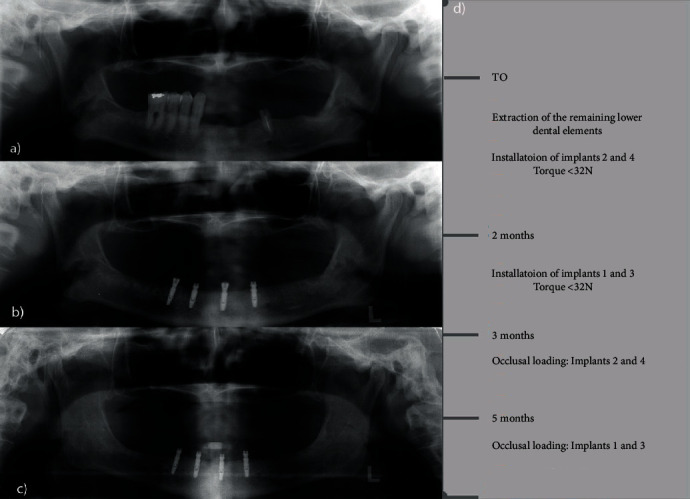
(a) Preoperative panoramic X-rays; (b) panoramic X-rays with installation of implants 2 and 4 (components installed) and implants 1 and 3 (healing caps); (c) final condition after loading all implants; (d) surgical timeline.

**Figure 5:**
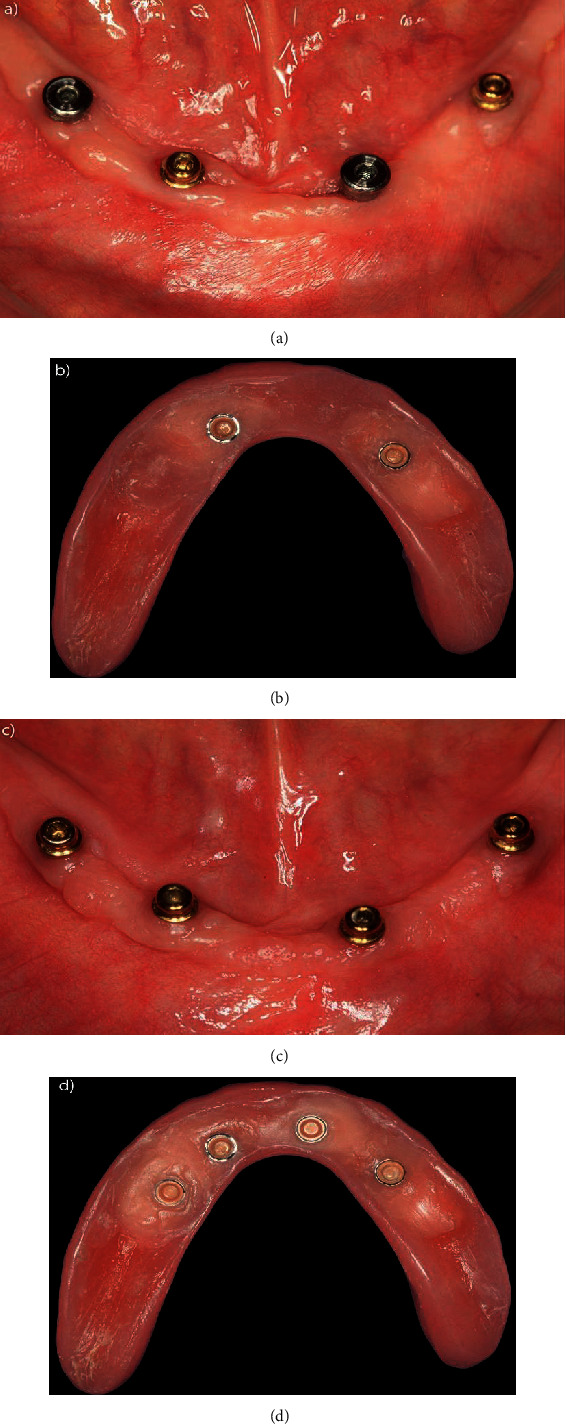
Clinical occlusal loading sequence: (a) occlusal view of Equator attachments connected to implants 2 and 4 and healing caps connected to implants 1 and 3; (b) base of the prosthesis after capturing the O-rings of implants 2 and 4; (c) occlusal view after installation of the Equator attachments connected to implants 1 and 3; (d) prosthesis base after capturing.

Patient 2 preoperatively presented a Kennedy Class I modification I type mandibular arch. After extraction, the patient had an ovoid mandibular ridge with an asymmetric, rounded residual ridge with a high muscle insertion close to the ridge. In this case, we opted to install 4 implants due to the patient's young age and request for high retention. However, because the patient's lower teeth were extracted at the time of surgery, adequate bone beds for all 4 implants were initially unavailable, so we opted to install implants 2 and 4 first, followed by installation of implants 1 and 3 after 2 months. The type of loading was conventional and progressive. Neither the first 2 implants (implants 2 and 4) nor the last 2 (implants 1 and 3) reached a minimum torque of 32 N, and all implants were thus loaded conventionally after 3 months of osseointegration.  Figure 4(a) shows the preoperative radiographic exams; the radiographic condition with installation of implants 2 and 4 with the components installed and implants 1 and 3 with healing caps is shown in Figure 4(b).  Figure 4(c) illustrates the final condition after loading all implants.  Figure 4(d) shows the surgical timeline.  Figure 5 summarizes the clinical occlusal loading sequence: Figures 5(a) and 5(b) show the capture of Equator attachments 2 and 4 three months after surgery, and Figures 5(c) and 5(d) show the capture of Equator attachemnts 1 and 3 three months after surgery 2.

### 2.3. Case 3

**Figure 6:**
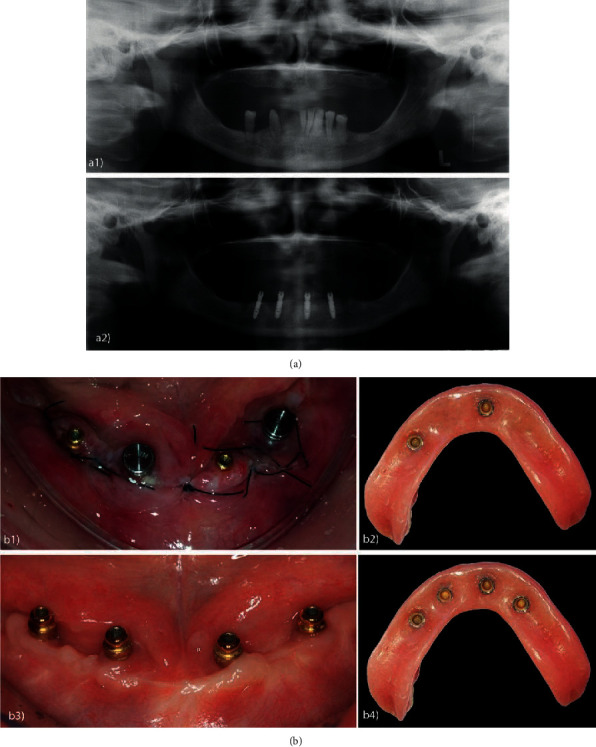
: (a) Panoramic X-rays: (a1) preoperative and (a2) immediate postoperative; (b) hybrid occlusal loading: (b1) occlusal view of the O-rings with cylinders in position for capture; (b2) view of the prosthesis base after capturing the O-rings with cylinders of implants 2 and 4; (b3) occlusal view of the attachments prior to capturing implants 1 and 3; (b4) view of the prosthesis base after final capturing.

Patient 3 preoperatively presented a Kennedy Class I, modification II type mandibular arch. After extraction, the patient had an ovoid mandibular ridge, with an asymmetric, knife edge-type residual ridge with a high muscle insertion close to the ridge. In this case, we opted to install 4 implants immediately after dental extractions in the lower arch. The bone availability and bone bed conditions at the time of surgery permitted to install all 4 implants simultaneously. However, unlike in case 2, patient 3 received hybrid loading: two implants reached torque > 32 N (implants 2 and 4) and received immediate occlusal loading, while the other two (implants 1 and 3) did not reach the minimum torque and received conventional loading. At the time of surgery, 4 Equatorattachments were installed but only two were immediately loaded.  Figure 6(a) shows the preoperative radiographic exams (Figure 6(a1)) and those made in the immediate postoperative period (Figure 6(a2)).  Figure 6(b) illustrates the adopted occlusal loading scheme: immediate loading for implants 2 and 4 (Figures 6(b1) and 6(b2)) and conventional loading for implants 1 and 3 (Figures 6(b3) and 6(b4)).

### 2.4. Monitoring of Clinical and Patient-Centered Variables

Assessments of the masticatory function and oral health-related quality of life (OHRQoL) were carried out before installation and after 1 year of MO use. All individuals had high expectations for the treatment and reported complaints related to masticatory function and showed dissatisfaction with their own oral health and quality of life. Peri-implant health and marginal bone loss (MBL) around the implants was monitored to verify the presence of differences in these outcomes in cases with different numbers of installed implants.

Masticatory function was assessed via the swallowing threshold test wherein individuals chewed a standardized portion of test food (3.7 g Optocal cubes) until they felt the desire to swallow [17]. Afterwards, the chewed material was expelled on a paper filter, dried at room temperature for 7 days, and sieved on a shaker with a sieve stack with sieve apertures between 5.6 and 0.5 mm. The material retained in each sieve was subsequently weighed, and the values were inserted in the Rosin-Rammler equation to calculate 2 parameters indicating the minimum opening of the sieve through which at least 50% of the chewed material would pass (ST_X50) and the homogeneity of the chewed particles (STB). In addition, the masticatory efficiency parameters ME5.6 and ME2.8 were calculated as the percentage of weight retained in the sieves with 5.6 and 2.8 mm apertures. The OHRQoL was assessed using the DIDL questionnaire [18] that divides the OHRQoL into 5 domains relating to the appearance of the prosthesis, pain during use, oral comfort, general performance, and eating and chewing.

## 3. Clinical and Functional Results after the MO Loading

**Figure 7:**
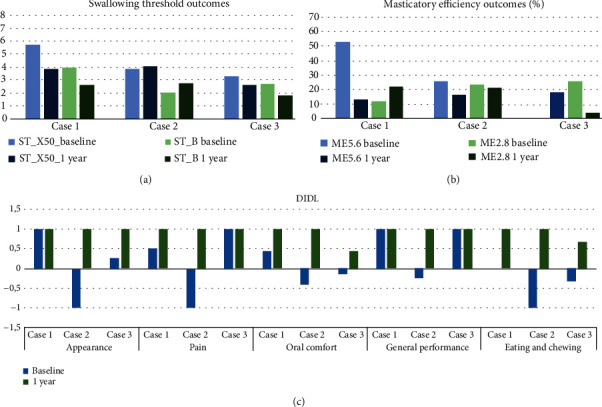
(a) Swallowing threshold outcomes (in mm). (b) Masticatory efficiency outcomes (in %). (c) Oral health-related quality of life outcomes (DIDL questionnaire scores; higher is better).

All patients showed signs of healthy peri-implant conditions during healing and postloading. The patient in case 2 showed the greatest peri-implant bone loss (Table 2). The ST_X50 values for 2 out of 3 patients decreased, indicating that food trituration improved after 1 year of MO use regardless of the number of implants installed as retainers. The trituration capacity of patient 2 deteriorated slightly (ST_X50 + 4.30%) as well as the homogeneous particle size distributions after 1 year of MO use, as indicated by an increase of 33.82% in STB (Figure 7(a)). The masticatory efficiency measured by ME5.6 improved in all cases, with the exception of case 3 (Figure 7(b)). All cases reported self-perceived OHRQoL improvements in the oral comfort and﻿ eating and chewing domain (Figure 7(c)).

## 4. Discussion

Treatment with dental implants has become an integral part of modern dentistry, even in challenging situations, such as completely edentulous elderly patients with systemic impairments, long periods of edentulism, mandibular bone atrophy, and/or poor-quality bone. The specialized literature shows that MO use for rehabilitation of totally edentulous individuals with low bone availability is well established and has satisfactory success and survival rates alongside a major positive impact on patients' quality of life [10, 19, 20]. MO treatment benefits from fast, low-cost minimally invasive techniques, and these have been undergoing innovations over the years [4]. Narrow diameter implant (NDI) systems designed for rehabilitation of patients with low bone availability have recently been studied to determine short- and long-term predictability and the maintenance regime required. A systematic review [7] showed that NDI have a 98% survival rate and a 96% success rate in up to 4 years, indicating that NDI are a safe treatment option with excellent long-term predictability and are thus a viable option to improve retention and stability of complete dentures [21].

Studies of this research group that investigated the Facility-Equator NDI system have observed that patients show a fast and significant increase in masticatory function 30 days after installation of 2 implant-retained MO; after three months, there is an OHRQoL improvement in the functional, oral comfort, and psychosocial domains [10, 11]. We also investigated the influence of anteroposterior discrepancies on the masticatory function and found that only Class II patients still had masticatory difficulties after 1 year of MO use [3]. While bone remodeling values of −0.06 ± 0.64 mm indicate that MBL 1 year after implant placement was similar to the MBL immediately after implant placement, dislodgements of the prosthetic and surgical components were the most common prosthetic complications [8]. In addition, monitoring clinical peri-implant health-related parameters, smoking habit, and time since edentulism in patients rehabilitated with this NDI system is important, as they can predict implant success rates [22].

The biological performance of the Facility-Equator system in the early and late osseointegration period was also evaluated according to conventional loading (CL) and immediate loading (IL) [23]. During the initial 3-month period, IL implants were associated with a smaller and thus more favorable probing depth than CL implants, while inflammatory markers such as TNF-*α* and IL-1*β* were more stable in the CC group [23]. The drilling depth remained lower in the IL group until 1 year after loading, and no significant differences in MBL, masticatory function, and OHRQoL were found between the CL and IL groups in this period. However, IL patients require more time to acquire similar chewing performance than CL patients. The survival rates and number of prosthetic intercurrences were also similar in both groups: 90% in the CL group (2 losses) and 85% in the IL group (3 losses); 33 prosthetic events occurred in the CL group and 23 in the IL group, mainly Equator dislodgements, followed by replacement of the prosthetic abutment [24].

There is currently a tendency to increase the number of implants used to retain MO, as some studies have shown that MO retained by 2 implants between the mental foramen have biomechanical disadvantages compared to MO retained by 3 or 4 implants, as 2 implants permit anteroposterior movements of the prosthesis, increasing the tension around the implants [13], accelerating MBL and bone loss in the posterior region of the ridge and reducing the masticatory capacity and satisfaction with the rehabilitation [12, 13, 25]. However, individual results can deviate strongly from such general tendencies. Cases 1 and 3 all show marked improvements in both food trituration and food homogenization and in the percentage of retention in the 5.6 and 2.8 mm sieves. Meanwhile, case 2 with MO retained by 4 progressively loaded implants experienced a slight deterioration (by 4.30%) for the ST_X50 and 33.82% for the STB and 84.51% in ME2.8. In addition, there was a more marked MBL in all implants in case 2, although this patient reported improvements in satisfaction and OHRQoL. The observed MBL in case 2 is unexpected since several studies [13, 14, 26] demonstrate that a greater number of implants guarantees lower tensions in the bone tissue around the implants and consequently less peri-implant bone loss is expected. A finite element study [13] showed that a greater number of implants result in more bone deformation around these implants, resulting in greater tension in the cortical bone around them, presenting greater MBL, while the masticatory force mostly affects the mucosa in cases with a lower number of implants. Such a process could account for the observed MBL in case 2. The lack of improvement in masticatory function in case 2 could be attributed to faster chewing by this patient, enabled by the greater retention and stability. Indeed, van der Bilt [27] has shown that a smaller number of cycles and time can directly interfere with the grinding of food (ST_X50), as individuals tend to chew less often and want to swallow the food faster. In addition, subjects with good masticatory function do not always swallow food after a smaller number of cycles, as the ST is partly influenced by the physiology of the individuals (masticatory muscles) and by the social context [27]. When chewing faster, with fewer cycles and due to the masticatory musculature being used only for this purpose and no longer to stabilize the prosthesis in the mouth, individuals perceive themselves as good chewers, thus guaranteeing improvements in the mastication and oral comfort domains, regardless of the number of implants.

It is clear that MO retained by 3 or 4 NDI loaded with conventional, immediate, progressive, and hybrid loading protocols can promote improvements in masticatory function, OHRQoL, and MBL of totally edentulous patients with limited bone availability. However, since a longitudinal clinical study [28] demonstrated that MO retained by three implants have similar survival rates as those retained by four implants and because our case report illustrates that using 4 implants can lead to suboptimal results in some cases, we emphasize that using 2 and 3 implants appears to reduce retention and stability problems of complete dentures sufficiently in many cases [9–12], while avoiding overtreatment and reducing financial costs for the patient, thus constituting treatment options with better cost-effectiveness.

The strengths of these approaches include the predictability of the treatment using narrow diameter implants that is attributed to (i) detailed case planning that took into account the clinical conditions along with the patient's preference and expectations and (ii) consistent clinical, functional, and OHRQoL improvements, regardless of the number of implants. We emphasize that MO retained by a smaller number of implants can fulfill their role adequately, with positive bone tissue, chewing, and quality of life responses, as in our case with 3 NDI. Therefore, this option can be strongly indicated for individuals with low bone availability and a reduced budget. The limitations for the proposed approaches are (i) the financial aspect: both the initial costs and the maintenance costs scale with the number of implants (e.g., O-rings require replacement annually) and (ii) with progressive aging and cognitive and motor decline, the increased retention of MO promoted by the greater number of implants can result in difficulties removing them; conversion to retention by 2 implants is recommended in these cases.

Finally, narrow diameter implants can be successfully used as MO retainers in diverse clinical scenarios where mandibular ridge atrophy is present. The available treatment options in these cases include the use of 3 or 4 implants and adopting conventional, conventional progressive, or hybrid loading, illustrating the versatility of the available clinical options. We recommend clinicians to analyze each case individually, taking into account the clinical conditions and the patient's preferences, together with the literature recommendations. Furthermore, we suggest that MO retained by 3 NDI can be indicated in the clinical routine as an optimal option when the patients wish increased retention and stability at a lower budget.

## Figures and Tables

**Table 1:** Sociodemographic, clinical, radiographic, and financial data.

Case	1	2	3
Gender	Masculine	Feminine	Feminine
Age	68	59	50
Ethnicity	White	White	White
Profession	Retired (merchant)	Housewife	Guard
Marital status	Married	Widow	Married
Time since mandibular edentulism	1	0	0
Time since maxillary edentulism	5	2	4
Distance between foramens	61.9	52.2	43.6
Cawood & Howell atrophy (anterior height/posterior height)	Nonatrophic (32.5 mm/29.9 mm)	Nonatrophic (25.5 mm/21.3 mm)	Nonatrophic (25.0 mm/19.1 mm)
Wical atrophy (mental foramen evaluation)	Atrophic	Atrophic	Atrophic
Kapur atrophy (clinical evaluation)	Nonatrophic	Atrophic	Atrophic
Main complaint for MO option	Lack of retention and stability of the lower prosthesis	Dissatisfaction with the appearance of the teeth and lack of retention of the lower prosthesis	Dissatisfaction due to pain and discomfort caused by lower prosthesis
Comorbidity	Alcoholic for 20 years and smoker for 9 years	—	Arterial hypertension
Transmucosal height	3.5 mm—left and midline; 2.5—right	2.5 mm—right and left	4.5 mm—right and left
Materials used	3 implants + 3 healing caps + 3 Equator	4 implants + 4 healing caps + 4 Equator	4 implants + 2 healing caps + 4 Equator
Minimum cost (BRL)	720.00	960.00	920.00

**Table 2:** Marginal bone loss by case, number of implants (I), and type of loading. Abbreviations: IL: immediate loading; CL: conventional loading; HL: hybrid loading.

		Baseline	1 year
Case 1-3I CL	I2	0.00	0.00
	I1	-0.12	1.13
	I2	0.00	1.70
	I3	0.00	1.57
Case 2-4I CL	I1	0.00	-0.62
	I2	0.00	-0.74
	I3	0.00	-0.45
	I4	0.00	-1.94
Case 3-4I HL	I1	0.00	0.00
	I2	0.00	-0.30
	I3	0.00	0.00
	I4	0.00	0.00
